# Implication of Different HIV-1 Genes in the Modulation of Autophagy

**DOI:** 10.3390/v9120389

**Published:** 2017-12-18

**Authors:** Zhenlong Liu, Yong Xiao, Cynthia Torresilla, Éric Rassart, Benoit Barbeau

**Affiliations:** Département des sciences biologiques and Centre de recherche BioMed, Université du Québec à Montréal, Montreal, QC H2X 3X8, Canada; liu.zhenlong@courrier.uqam.ca (Z.L.); yxiao37@yahoo.ca (Y.X.); cynthia.torresilla@gmail.com (C.T.); rassart.eric@uqam.ca (É.R.)

**Keywords:** HIV-1, autophagy, viral protein

## Abstract

Autophagy is a complex cellular degradation pathway, which plays important roles in the regulation of several developmental processes, cellular stress responses, and immune responses induced by pathogens. A number of studies have previously demonstrated that HIV-1 was capable of altering the regulation of autophagy and that this biological process could be induced in uninfected and infected cells. Furthermore, previous reports have indicated that the involvement of HIV-1 in autophagy regulation is a complex phenomenon and that different viral proteins are contributing in its modulation upon viral infection. Herein, we review the recent literature over the complex crosstalk of the autophagy pathway and HIV-1, with a particular focus on HIV-1 viral proteins, which have been shown to modulate autophagy.

## 1. Introduction

The dysregulation of autophagy is associated to many diseases such as cancer [1], heart dysfunction [2], neurodegenerative disorders [3], and infectious diseases [4,5]. Autophagy is an importantly regulated catabolic process, which degrades and clears excess or damaged proteins, organelles, and pathogen-derived proteins, and allows maintaining cellular nutrient levels during conditions of starvation [6]. In mammals, there are three distinct autophagic pathways: macroautophagy, microautophagy, and chaperone-mediated autophagy (CMA) [7]. These three autophagy pathways have distinct features, which allow each to degrade specific cellular components by distinctive machineries. The first pathway is termed macroautophagy and degrades substrates of diverse origins packaged into a double-membrane structure termed the autophagosome, which eventually fuses with lysosomes. The second pathway, microautophagy, directly captures target cytosolic substrates through the invagination of membranes into the lysosome. The third pathway, referred to as the chaperone-mediated autophagy, leads to the degradation of proteins harboring the KFERQ domain, which allows them to interact with the lysosomal membrane protein LAMP2A (Lysosome-associated membrane protein 2) and the HSC70 (Heat shock cognate 71 kDa protein) chaperone [8]. In addition to these autophagy pathways, a fourth pathway has also been described, which selectively degrades defective mitochondria and is consequently termed mitophagy.

The major form of autophagy is macroautophagy and will be hereafter referred to as “autophagy” for simplicity. Ever since autophagy has been first reported in 1962 [9], autophagy has been highlighted as a pathway in which degradation is mediated by the autolysosomes, which are formed by the fusion of autophagosomes to lysosomes. Non-essential and excess cellular components from the endoplasmic reticulum (ER), the Golgi, mitochondria, or endosomes are thus recruited to the double-membrane autophagosome structure and then digested by lysosomal acid hydrolases. These cellular constituents include dysfunctional cellular organelles, protein aggregates, misfolded proteins, and even invasive pathogens such as viruses [10]. An important number of previous studies have indicated that HIV infection can trigger and restrict autophagy, although data show important differences in the outcome of the infection, which is dependent on the studied cell type [11]. The aim of this review is to describe and summarize the relationship between autophagy and HIV and how the process of autophagy is altered during HIV infection. This information will be essential in guiding industries and researchers toward appropriate development of new drug designs of vaccines.

## 2. The Molecular Machinery of Autophagy

**Figure 1:**
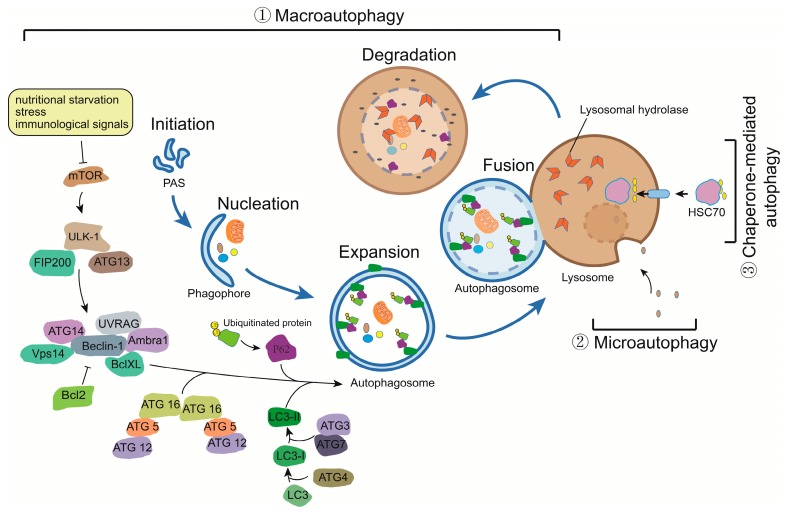
Three types of autophagy and regulatory mechanisms. Three types of autophagy are presented: ① macroautophagy, ② microautophagy, and ③ chaperone-mediated autophagy. Macroautophagy is divided in five steps: initiation, nucleation, expansion, fusion and degradation. Nutritional starvation, stress, and immunological signals initiate autophagy via inhibition of mTOR, which act as suppressors of the autophagy pathway through activation of the ULK1 (Unc-51-like kinase 1) complex. Nucleation and autophagosome formation further requires the Beclin-1-Atg14L-Vps34 complex (producing PI3P (Phosphatidylinositol-3-phosphate)) and two ubiquitin-like conjugation systems: Atg12-Atg5-Atg16L1 and PE (Phosphatidylethanolamine) conjugation of LC3-I forming LC3-II. After the fusion of autophagosome to lysosome, lysosomal hydrolases degrade the content of the autophagosome. T bars indicate inhibition of activity or complex.

Autophagy is a cellular pathway, which is highly conserved in all eukaryotes and regulated by more than thirty identified autophagy-related genes [12] (Figure 1). The autophagy pathway includes three distinct stages: (1) initiation by inactivation of the mammalian target of rapamycin (mTOR), an important inhibitor of autophagy; (2) formation of the autophagosome involving the binding of the LC3II (LC3 (Microtubule-associated protein 1A/1B-light chain 3)-phosphatidylethanolamine conjugate) autophagy factor to the double-membrane vesicle and cytoplasmic constituents, such as subcellular organelles and microbial pathogens engulfed into autophagosome; and (3) autophagosome-lysosome fusion, which leads to the degradation of targeted substrates [13,14].

Induction of autophagy initiates from the inhibition of mTOR activity by a variety of stress and immunological signals [15]. There are two typical complexes that are involved in the tight regulation of autophagy: mTOR complex 1 (mTORC1) and mTOR complex 2 (mTORC2) [16]. Two important upstream kinase regulators are implicated in this regulation: MAP4K3 (mitogen-activated protein kinase kinase kinase kinase 3) [17] and hVps34 (the human orthologue of yeast vacuolar protein sorting 34, Vps34) also referred to as PI3KIII (phosphoinositide 3-kinase class III) [18]. ULK1 (Unc-51-like kinase 1), a serine/threonine protein kinase (the orthologue of yeast Atg1) initiates autophagy by phosphorylating Beclin-1, which leads to the disassociation of Beclin-1 from Bcl-2, another inhibitor of autophagy [18,19,20]. Subsequently, Beclin-1 activates the lipid kinase Vps34 to form the Beclin-1-Atg14L-Vps34 complex thereby producing phosphatidylinositol-3-phosphate (PI3P) [21,22] and providing a membrane platform for the assembly of autophagic proteins, which sequentially regulates membrane trafficking and initiation of the nucleation and elongation of the autophagosomes [23]. In the next step, during the elongation stage of autophagy, two ubiquitin-like conjugation systems are required: Atg12-Atg5-Atg16L1 (catalyzed by Atg7 and Atg10) and the phosphatidylethanolamine (PE)-conjugated LC3-I forming LC3-II (catalyzed by Atg4, Atg7, and Atg3). LC3II is embedded in the autophagosome membrane and regulates its formation and maturation. This important component of the autophagosome is often referred to as a classical autophagy marker [24]. At the last step of autophagy, the small GTPase Rab7 supports the fusion of autophagosomes and lysosomes, leading to formation of autolysosomes and ensuing degradation of their captured contents [25,26].

Unlike general autophagy, selective autophagy assembles and degrades substrates specifically recognized by autophagy adaptors (sequestosome-1-like receptors), such as p62/SQSTM1 (Sequestosome-1), Optineurin and NDP52 (Nuclear dot protein 52 kDa) [27]. Importantly, p62/SQSTM1 has multiple functional domains, including a Phox1 and Bem1p (PB1) domain (aggregating p62/SQSTM1 multimers), an LC3-interacting region (LIR, recognizing and interacting with LC3-II, allowing the packaging of p62/SQSTM1 cargos), and a Ubiquitin-associated (UBA) domain (recognizing and interacting with ubiquitinated substrates) [28]. Other domains are also present in p62/SQSTM1, such as nuclear shuttling signals, a Keap1-interacting region (KIR), and a Tumour necrosis factor receptor-associated factor 6 (TRAF6) binding domain, which acts as a signaling hub to regulate mTORC1 translocation and activation to lysosome by interaction of p62/SQSTM1 and TRAF6 [29,30]. Importantly, autophagosome-packaged p62/SQSTM1 and its cargo are subsequently degraded, which makes p62/SQSTM1 another marker to monitor autophagy.

## 3. A Crosstalk between Autophagy and HIV-1

**Figure 2:**
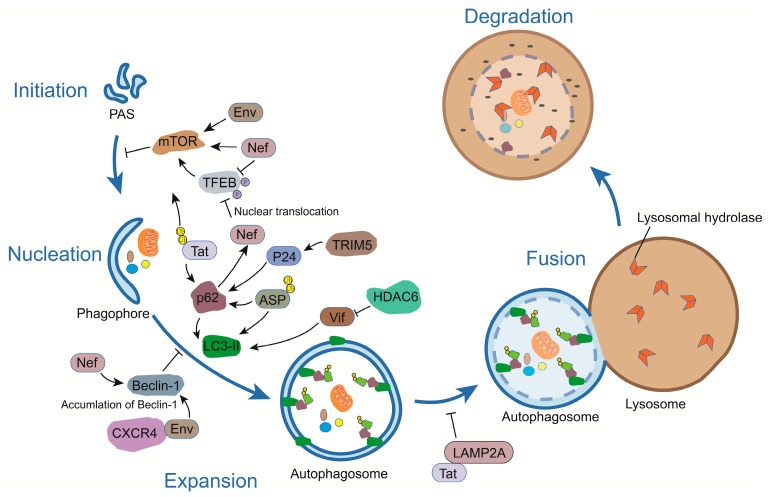
The link between HIV-encoded proteins and the autophagy pathway. Different HIV-1 proteins modulate autophagy and these are exemplified herein. While HIV-1 Env and ASP (Antisense protein) induce autophagy, the viral Nef protein restricts this pathway by activating mTOR or inhibiting Transcription factor EB (TFEB) phosphorylation and binding with Beclin-1. Tat is another viral protein which blocks autophagy by interacting with LAMP2A. Certain viral proteins, such as Tat (in its ubiquitinated form), p24, and ASP, can also be targeted by p62-mediated autophagy. T bars indicate inhibition of activity or complex.

The influence of viral proteins on the activation of the autophagy machinery has been illustrated in numerous studies using different viruses and has shown high levels of variation due to differences in cell types and cellular environments [4,11,31,32]. These reports have provided important information as to the complexity of autophagy and how different steps can be targeted and functionally altered by various viral proteins. Interestingly, certain viruses are known to use autophagic membranes to maximize their replication, while certain viruses escape autophagy-induced degradation by hijacking Atgs and restricting autophagy [4]. Due to the requirement of autophagy for the early replication steps of HIV-1, this virus has developed many strategies to escape the antiviral properties of this biological process. A genomic screen has, in fact, identified a number of autophagy-related host factors, including Atg7, Atg8, Atg12, and Atg16L2, which were essential for HIV-1 infection [33]. Several excellent reviews have summarized the complex relationship between autophagy and HIV infection [4,11,31,32] and, in the following sections, we will discuss and update recent reports on the association between autophagy and HIV-1 with a focus on the implicated viral proteins (Figure 2).

### 3.1. Env

The HIV-1 *env* gene encodes an envelope glycoprotein precursor, which, upon cleavage by the furin cellular protease, results in the generation of gp120 and the gp41 transmembrane glycoprotein [34]. During HIV-1 entry, the gp120 subunit at the surface of the virus binds to the CD4 receptor and a co-receptor, e.g., mainly CCR5 (C-C chemokine receptor type 5) or CXCR4 (C-X-C chemokine receptor type 4), depending on the viral strain (termed R5 or X4, respectively). Interestingly, it has been reported that gp120 can induce apoptosis of uninfected bystander T cells expressing CD4 and CXCR4 or CCR5 through various mechanisms [35]. An important publication has subsequently demonstrated that this apoptosis-associated phenomenon was also paralleled by the accumulation of Beclin 1 in uninfected CD4+ T lymphocytes via CXCR4 binding and autophagy induction [36]. This report further highlighted that CD4 signaling and p56lck were not required and that autophagy was necessary for apoptosis to be induced. In a subsequent study, the team of Biard-Piechaczyk demonstrated that CXCR4 signaling was not implicated in Env-induced autophagy, but was highly dependent on the gp41 fusion domain [37]. Autophagy can also be induced by R5 virus-derived Env upon binding to uninfected CCR5-expressing CD4+ T cells but is inhibited in CD4+ T cells infected by either X4 or R5 strains [38]. Interestingly, autophagy is not similarly induced in uninfected macrophages following exposure to viral particles, despite being positive for the presence of autophagosomes. However, in infected macrophages, viral replication is being favored by induced autophagy (see below). At a more physiologically-relevant level, Zhou et al. found that the levels of Beclin 1, Atg5, Atg7, and LC3II increased in postmortem brains presenting HIV-1 encephalitis compared with HIV-1 patients without encephalitis. Additionally, these authors confirmed that, in the neuroblastoma SK-N-SH cell line, overexpression of both CXCR4- or CCR5-specific gp120 increased the presence of these autophagy markers. This study thereby further suggests that HIV-1 gp120 induces autophagy in neuron cells, and that the induction of autophagy might be related to the pathogenesis of neuroAIDS [39].

The association between the Env protein, mostly the gp41 subunit and autophagy, has been very well established. However, more studies are direly needed to better understand the mechanism by which it operates in a cell type-dependent manner.

### 3.2. Gag

The Gag polyprotein is cleaved in various polypeptides known as the matrix (MA), the capsid (CA), the nucleocapsid (NC), the spacer peptides SP1 and SP2, and p6, which subsequently controls viral assembly and viral budding [40]. Kyei et al. showed that, during the early steps of autophagy, the Gag­derived polypeptides were found to interact with LC3-II in macrophages, as determined by confocal microscopy and immunoprecipitation experiments [41]. They provided additional evidence that Gag processing was augmented when autophagy was induced, demonstrating that this biological process led to maximal viral replication in infected macrophages.

An important characteristic of autophagy is that it also plays an important role in innate and adaptive immunity, and that viruses have evolved mechanisms to counteract the process by which they are subjected to degradation by the autophagolysosome. Of note, half of the tripartite motif [42] protein family, harboring known HIV restriction factors, has been identified as regulators and as cargo receptors of autophagy. In this respect, Mandell et al. provided interesting evidence that TRIM proteins can interact with and regulate ULK1 and Beclin1, resulting in the formation of a multimolecular complex and that it acted as a cargo receptor mediating its viral restrictive function through autophagy-dependent degradation of the viral particle [43,44]. In fact, TRIM5a, an important inhibitor of HIV-1 replication [45,46], restricts HIV-1 replication by binding p62/SQSTM1 and by recognizing and targeting HIV-1 Gag p24 for selective autophagy degradation [44,47]. Of further importance, TRIM21 regulates autophagy by interacting with IRF3 (IFN regulatory factor 3) and modulating its stability during virus infection [48].

The Gag polyprotein is an important target of autophagy, but HIV-1 seems to have taken advantage of Gag targeting for its replication, at least in macrophages. It still needs to be determined why this process seems to be selective to certain cell types.

### 3.3. Tat

Tat is one of well-known regulatory proteins required for HIV-1 replication. Its main function is the upregulation of transcription from the 5′ Long terminal repeat (LTR)-containing promoter through the formation on a complex with the TAR hairpin region of nascent RNA transcripts [49]. Due to its capacity to strongly modulate viral transcription, Tat also has an important impact on viral latency [50]. Tat is differently targeted by processes, which alter its abundance. Ubiquitination is a post-translational modification that has been shown to target this viral protein. Indeed, ubiquitination of Tat leads to proteasome-dependent degradation although Bres et al. have also shown that a different type of ubiquitination of Tat promotes a higher level of LTR transactivation [51,52]. A recent study has shown that autophagy also selectively targets Tat through a ubiquitin-independent interaction of Tat and p62/SQSTM1 in CD4+ T lymphocytes. Further evidence in this study, however, suggests that Tat degradation is eventually blocked to allow viral replication [53]. In another study focusing on patients suffering from HIV-associated neurocognitive disorder (HAND), Tat was shown to induce a decrease in the abundance of the autophagy markers LC3II andp62/SQSTM1 associated to membrane in neurons [54]. The authors further demonstrated that Tat could bind to the lysosomal-associated membrane protein 2A (LAMP2A) both in vivo and in vitro, thereby possibly leading to an upregulation of the fusion of autophagosomes with lysosomes.

The limited number of studies linking Tat to autophagy is of interest, but more data and evidence toward the impact of autophagy on Tat stability in infected cells will be required.

### 3.4. Nef

The accessory protein Nef (Negative regulatory factor) plays fundamental roles in host membrane trafficking and receptor downregulation during virus replication [55]. Recent studies have revealed that the transmembrane factor SERINC5 (serine incorporator 5), which inhibits HIV-1 infectivity, can be redirected to the Rab7-positive endosomal compartment by Nef thereby providing the first mechanism by which this viral protein can increase HIV-1 infectivity in a host-cell-dependent manner [56,57]. Nef has also been shown to be an important modulator of autophagy and in fact inhibits late steps in order to avoid degradation of viral particles. Detailed studies have revealed that Nef binds to the conserved domain of the autophagy regulatory factor Beclin-1, which competes for the binding of glioma-associated oncogene pathogenesis-related 2 (GLIPR2). This potentially leads to the inhibition of autolysosome maturation observed in macrophages and eventually to the escape of viral capsids from autophagy-mediated degradation [41,58]. Another report has demonstrated that, during early stages of HIV-1 replication in macrophages, autophagy is induced by HIV-1, through the toll-like receptor 8 (TLR8) which, in turn, depends on Beclin1-dependent dephosphorylation and nuclear translocation of the transcription factor EB (TFEB). Upon active HIV-1 replication, autophagy is restricted through mTOR activation and phosphorylation/cytoplasmic sequestration of TFEB mediated by the interaction of Nef and Beclin1 [59]. Interestingly, studies in astrocytes have revealed that high expression of Nef typically observed in astrocytes of HIV-1-infected brains increased LC3II and p62/SQSTM1 levels in these cells and that this accumulation strongly correlated with blocking of the fusion of autophagosome to lysosome and escape of viral degradation [60,61].

Based on previous studies, Nef is a clear negative modulator of autophagy and is determinant in allowing HIV-1 replication in otherwise hostile cellular environment. Whether this is the only HIV-1 protein capable of inhibiting this degradative pathway remains to be determined.

### 3.5. Vif

Vif (Viral infectivity factor) is another HIV-1 accessory protein [62]. Its main function is to promote ubiquitination and degradation of the cytidine deaminase APOBEC3G (A3G), thereby inhibiting A3G antiviral activity [63]. In a study by Borel et al. [64], a new function was assigned to Vif in relation to autophagy. It was reported that Vif could interact with LC3II independently of the presence of APOBEC3G. Furthermore, autophagy was induced in NL4.3ΔVif-infected cells, while Vif overexpression led to autophagy inhibition. Despite its potential inhibitory role, Vif can nonetheless be a substrate toward autophagy. Indeed, the HDAC6/A3G complex can positively regulate autophagy and induce subsequent degradation of the Vif protein [65]. Accordingly, HDAC6 interacts with and promotes Vif degradation, and thereby blocks Vif-mediated A3G degradation [65].

This HIV-1 protein has been shown to be a potential inhibitor, but also a target to autophagy. In future studies, it will be interesting to assess how important this protein is in the modulation of autophagy in infected cells.

### 3.6. Vpu

Vpu, another HIV-1 accessory protein, enhances virion budding by targeting human CD4 and Tetherin/BST2 (bone marrow stromal cell antigen 2) to proteasome degradation. Mechanistically, tetherin blocks HIV-1 particle release and, therefore, leads to the formation of aggregated viral particles at the host cell membrane, while Vpu mediates interaction of either CD4 or BST2 to BTRC (βTrCP-1), a substrate recognition subunit of the Skp1/Cullin/F-box E3 ubiquitin ligase, leading to their ubiquitination and subsequent proteasomal degradation [66]. A recent study by Madjo et al. indicated that Vpu selectively binds to the autophagy factor LC3C, which leads to the removal of BST2 from the HIV-1 budding region and could thereby counteract BST2 restriction [67].

Vpu provides a new mean by which autophagy acts on HIV-1 replication, although more studies will be needed to address its association to this biological process.

### 3.7. ASP

Antisense transcription has been demonstrated to be important in the modulation of HIV-1 gene expression but has further been proposed to encode a protein termed antisense protein (ASP), whose existence has recently been supported by detailed in silico analyses [68,69]. Other studies further revealed that ASP could be detected by in vitro expression and that specific antibodies and cytotoxic CD8+ T lymphocyte (CTL) response were detected in patients [70,71,72,73,74,75]. The existence of ASP has been a source of controversy and is partly associated to its difficult detection, although its early detection has been possible through electron microscopy [76]. In our recent study, we have demonstrated that expression of ASP induced autophagy, potentially through its capacity to form multimers [71]. We have further shown that in monocytic cells, ASP-induced autophagy led to an increase in HIV-1 replication, which concurs with a previous observation by Kyei et al. [41].

It is very likely that several reports on this protein will emerge in in the upcoming years and will further shed light on its implication in the control of autophagy and, consequently, HIV-1 replication.

## 4. Conclusions

Since the early study showing that the envelope protein could impact uninfected cells, the association between HIV-1 infection and autophagy has been strongly studied for over 10 years. Numerous studies have since then revealed that several viral proteins are capable of modulating autophagy to favor HIV-1 replication although these effects appear to be context-dependent. Like for other viruses, HIV-1 needs to modulate autophagy in order to replicate itself in infected cells and to alter immune responses. As it has been clearly shown, the modulation of autophagy by HIV-1 is very complex and involves many viral proteins. Future studies aimed at determining the mechanism behind HIV-1-mediated regulation of autophagy will be important and need to be undertaken in different targeted cells and with HIV-1 viruses from different clades. A focus on recently implicated viral protein, such as Vpu and ASP will also be required. The knowledge acquired from these studies will lead to a better understanding of HIV-1 replication and might lead to the identification of new interesting targets for future antiretroviral treatments.
